# Cardiovascular Effects of the Essential Oil of* Croton argyrophylloides* in Normotensive Rats: Role of the Autonomic Nervous System

**DOI:** 10.1155/2016/4106502

**Published:** 2016-11-13

**Authors:** Thayane Rebeca Alves-Santos, Rodrigo José Bezerra de Siqueira, Gloria Pinto Duarte, Saad Lahlou

**Affiliations:** ^1^Department of Physiology and Pharmacology, Federal University of Pernambuco, Recife, PE, Brazil; ^2^Department of Physiology and Pharmacology, Federal University of Ceará, Fortaleza, CE, Brazil

## Abstract

Cardiovascular effects of the essential oil of* Croton argyrophylloides* Muell. Arg. (EOCA) were investigated in normotensive rats. In saline-pretreated anesthetized or conscious rats, intravenous (i.v.) injection of the EOCA induced dose-dependent hypotension. Dose-dependent tachycardia was observed only in conscious rats. In anesthetized rats, cervical bivagotomy failed to enhance EOCA-induced hypotension but unmasked significant bradycardia. In conscious rats, i.v. pretreatment with methylatropine, but not with atenolol or L-NAME, reduced both hypotensive and tachycardiac responses to EOCA. However, hexamethonium pretreatment reverted the EOCA-induced tachycardia into significant bradycardia without affecting the hypotension. In aortic ring preparations precontracted with phenylephrine, EOCA induced a concentration-dependent relaxation that was significantly reduced by vascular endothelium removal and pretreatment with atropine, indomethacin, or glibenclamide but remained unaffected by pretreatment with L-NAME or TEA. It is concluded that i.v. treatment with EOAC decreased blood pressure probably through an active vascular relaxation rather than withdrawal of sympathetic tone. Muscarinic receptor stimulation, liberation of the endothelium-derived prostacyclin, and opening KATP channels are partially involved in the aortic relaxation induced by EOCA and in turn in the mediation of EOCA-induced hypotension. EOCA-induced tachycardia in conscious rats appears to be mediated reflexly through inhibition of vagal drive to the heart.

## 1. Introduction

*Croton argyrophylloides* Muell. Arg. (Euphorbiaceae) is a native bush from northeastern Brazil, mainly in the* caatinga* region, where it is popularly named as “marmeleiro prateado.” In folk medicine, a decoction is used as antidiabetic and also as baths for the treatment of venereal diseases [1]. Surprisingly, very little research has been carried out to examine the basic pharmacological properties of this plant. Antimicrobial activity has been reported for the first time for a diterpene isolated of* C. argyrophylloides*, the argyrophillic acid [2]. Essential oil of* C. argyrophylloides* (EOCA) demonstrated an interesting antifungal activity as it was effective only against the dermatophyte* Microsporum canis* [3], a finding suggesting that this essential oil could be a potential source for the synthesis of new phytotherapeutic to treat dermatophytosis. In addition, EOCA displays an antioxidant activity [4] and an interesting insecticidal activity against* Aedes aegypti* L. [5], two activities that were similar or even higher than that evoked by essential oils of other* Croton* species.

The genus* Croton* comprises about 1000 species, distributed mostly throughout the American continent. In Brazil, there are approximately 300* Croton* species predominantly in northeastern region [6]. It has been previously shown that essential oils from several plants of this genus bear two characteristics: they have a variety of pharmacological effects and small acute toxicity [5, 7, 8]. Amongst these effects, hypotensive action and antispasmodic activity of the essential oil of* C*.* nepetaefolius* [9–12],* C*.* zehntneri* [13–15], and* C*.* sonderianus* [16] on several types of smooth muscle have been previously reported by our research group.

Previously, we reported that EOCA was able to relax both a conduit artery and a resistance vascular bed preparation [8], an effect that could be responsible for a putative hypotensive action of this essential oil, as previously demonstrated for EOCN [9] and EOCZ [13, 14] in normotensive rats. As no information in the international literature is available regarding this hypothesis, the present study was undertaken to test such a possibility and also to elucidate the mechanisms underlying the vasorelaxant effects of EOCA.

## 2. Materials and Methods

### 2.1. Plant Material

Aerial parts of* C. argyrophylloides* were collected on April 2005 from the region of Viçosa do Ceará, a municipality of the state of Ceará, Brazil. Plant identification was confirmed by Dr. F. J. Abreu Matos (Laboratory of Natural Products, Federal University of Ceará) and by Dr. A. A. Fernandes (Department of Biology, Federal University of Ceará). A voucher specimen (number 32444) is deposited in the herbarium of Prisco Bezerra at that university.

### 2.2. Extraction and Chemical Analysis

The EOCA was kindly provided by Dr. J. H. Leal-Cardoso (Superior Institute of Biological Sciences, State University of Ceará, Fortaleza, Brazil). It was prepared from freshly chopped leaves by steam distillation and analyzed chemically as previously described [8]. Analytical conditions were as follows: EOCA analysis was performed by gas chromatography and mass spectrometry (GC/MS; model 6971; Hewlett-Packard, Palo Alto, CA, USA). The column was a dimethylpolysiloxane DB-1 fused silica capillary column (30 m × 0.25 mm; 0.1 *μ*m); the carrier was gas helium (1 mL/min); the injector temperature was 220°C; the detector temperature was 280°C; and the column temperature was increased from 50 to 180°C at 4°C/min and then from 180 to 250°C at 20°C/min. The mass spectra had an electronic impact at 70 eV. The chemical analysis of the sample of the EOCA used in the present study identified the following constituents (in % of oil weight): spathulenol (26.65%), caryophyllene oxide (13.13%), *β*-elemene (12.15%), *β*-caryophyllene (10.94%), *β*-germacrene (5.16%), 1,8 cineole (4.31%), allo-aromadrendene (3.41%), *β*-selinene (2.83%), *α*-humulene (2.56%), *α*-pinene (2.43%), aromadrendene (2.3%), sabinene (2.29%), and unidentified constituents (11.84%). These compounds were identified using a mass spectral library search and [^13^C]-nuclear magnetic resonance spectroscopy [17].

### 2.3. Solutions and Drugs

For* in vivo* experiments, EOCA was dissolved in Tween 80 (2%), brought to the chosen concentration with sterile isotonic saline under sonication. In both conscious and anesthetized rats, baseline MAP and HR values remained significantly unchanged following 4 successive bolus injections (100 *μ*L, i.v.) of the EOCA's vehicle separated by a 10 min period interval (data not shown), as previously reported [18, 19]. EOCA was injected manually as a bolus in a volume of 0.1 mL, followed by a 0.2 mL flush with physiological saline. Antagonists were dissolved in saline just before use and administered in a volume of 1 mL/kg body weight. For* in vitro* experiments, EOCA was first dissolved in Tween 80 (0.5%), made up with the perfusion medium and sonicated just before use. The perfusion medium used was fresh Krebs-Henseleit solution (KHS) buffer (pH 7.4) of the following composition (in mM): NaCl 118; KCl 4.7; NaHCO_3_ 25; CaCl_2_·2H_2_O 2.5; KH_2_PO_4_ 1.2; MgSO_4_·7H_2_O 1.2; glucose 11. Phenylephrine (PHE) hydrochloride, indomethacin, acetylcholine (ACh) chloride, tetraethylammonium chloride (TEA), glibenclamide and* N*-*ω*-nitro-l-arginine methyl ester (L-NAME) hydrochloride were first dissolved in distilled water and were made up with KHS in order to achieve desired concentration in bath chamber. Drugs were purchased from Sigma (St. Louis, USA).

### 2.4. Animals

Male Wistar rats (280–320 g) were obtained from the central housing facility of the Federal University of Pernambuco, Recife, Brazil, and were kept under standard conditions (temperature at 22 ± 2°C; 12 h light/dark cycle and food and water* ad libitum*). All animals were cared for in compliance with the Guide for the Care and Use of Laboratory Animals, published by the US National Institutes of Health (NIH Publication 85-23, revised 1996; http://www.nap.edu/readingroom/books/labrats/index.html). All procedures described here were reviewed by and had prior approval from the local animal ethics committee.

### 2.5. *In Vivo* Experiments

#### 2.5.1. Catheterization Procedure

Rats were anesthetized with sodium pentobarbital (50 mg/kg, i.p.), and catheters (PE-10 fused PE-50) were implanted in the abdominal aorta (for blood pressure recording) and in the inferior vena cava (for drug administration) through the left femoral artery and vein, respectively, as previously described [13]. Postoperatively, rats received an intramuscular injection of penicillin (24,000 IU). They were housed individually in plastic cages and allowed to recover for 24 h before any circulatory experiments.

#### 2.5.2. Recording of Mean Arterial Pressure and Heart Rate

At the time of experiment, arterial catheter was coupled to a pressure transducer and baseline mean arterial pressure (MAP) and heart rate (HR) values were recorded on a Gilson model 5/6H (Medical Electronics Inc., Middletown, WI, USA), as previously described [13].

#### 2.5.3. Experimental Design and Protocols

In order to explore the cardiovascular responses to EOCA, the following protocol was adopted. Before each experiment, a period of 15–20 min was allowed to obtain a stable MAP and HR tracing. Injections were separated by 10 min intervals in order to avoid tachyphylaxis.

Doses of antagonists and EOCA were chosen according to those recommended in the literature and those employed in our previous investigations [13, 14, 18, 20–23], respectively. In a first series of experiments, a dose-effect relationship was determined in anesthetized rats which have been submitted (*n* = 6) or not (*n* = 5) to a cervical bilateral vagotomy. Each animal received a series of increasing bolus doses of EOCA (1, 5, 10, and 20 mg/kg, i.v.), and time course of the changes in MAP and HR was recorded. In a second series of experiments, the role of the autonomic nervous system and oxide nitric (NO) in the mediation of EOCA-induced cardiovascular changes was assessed in conscious, freely moving rats. Therefore, maximal changes in MAP and HR elicited by i.v. injections of increasing bolus (100 *μ*L) doses of EOCA (1–20 mg/kg) were determined in conscious rats, which had been pretreated intravenously 10 min earlier with either saline (1 mL/kg, *n* = 5), atenolol (1.5 mg/kg, *n* = 5), L-NAME (20 mg/kg, *n* = 6), hexamethonium (30 mg/kg, *n* = 5), or methylatropine (1 mg/kg, *n* = 7).

### 2.6. *In Vitro* Experiments

Experiments with aortic ring preparations were conducted in a separate set of rats according to a previously reported protocol [24]. Briefly, rats were sacrificed and thoracic aorta was removed and placed in cold oxygenated KHS buffer. Ring-like segments of this artery (3 mm in length) were obtained free of fat and connective tissue, and they were mounted between two steel hooks in 5 mL isolated tissue chambers containing gassed (95% O_2_ and 5% CO_2_) KHS, at 37°C, under a resting tension of 1 g, which was readjusted every 15 min during a 45 min equilibration period before drug administration. Isometric tension was recorded by using an isometric force displacement transducer (Grass Model FTO3, Quincy, MA, USA) connected to an acquisition system (PM-1000; CWE Inc., Akron, OH, USA). Vessels were initially exposed twice to 60 mM KCl to check their functional integrity. After 30 min, rings were contracted with PHE (0.1 *μ*M, a concentration that induces 50–70% of the contraction induced by KCl) and ACh (1 *μ*M) was then added to assess endothelium integrity. Sixty min later, a sustained contraction was induced again by PHE (1 *μ*M) and the effects of cumulative concentrations (1–1000 *μ*g/mL) of EOCA were studied in either endothelium-intact (*n* = 5) or endothelium-denuded (*n* = 5) aortic ring preparations. In order to assess the role of the cholinergic system, NO synthase, prostaglandins, or potassium channels in the effects of EOCA in PHE-contracted tissues, experiments were performed in endothelium-containing aortic ring preparations incubated for 30 min with atropine (1 *μ*M, *n* = 5), L-NAME (100 *μ*M, *n* = 5), indomethacin (10 *μ*M, *n* = 5), glibenclamide (100 *μ*M, *n* = 5), or TEA (5 mM, *n* = 5), respectively. Effects of vehicle at the same concentrations used to dissolve EOCA were also studied.

### 2.7. Statistical Analysis

All results are expressed as the mean ± SEM. Maximal changes in MAP and HR after each dose of the EOCA were expressed as a percentage of baseline values. For* in vitro* experiments, peak deflections measured the magnitude of the concentration-response curves, which was expressed as a percentage of PHE- (1 *μ*M) induced contractions. IC_50_ value, defined as the EOCA concentration (*μ*g/mL) required to produce a half maximum reduction of PHE-induced contractions, was used to evaluate vascular sensitivity to this essential oil. It was calculated by interpolation from semilogarithmic plots and was expressed as geometric mean [95% confidence interval]. Significance (*p* < 0.05) of results was assessed by paired Student's *t*-test, Mann–Whitney *U* test, and one- or two-way analysis of variance (ANOVA), followed by Dunnett's multiple comparison test when appropriate.

## 3. Results

### 3.1. *In Vivo* Experiments

**Figure 1:**
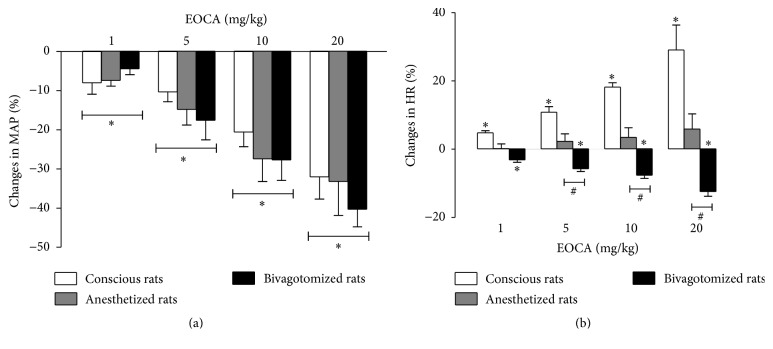
Maximal changes in mean aortic pressure (ΔMAP max, (a)) and heart rate (ΔHR max, (b)) elicited by intravenous (i.v.) injections of increasing bolus doses (1–20 mg/kg) of the essential oil of* Croton argyrophylloides* (EOCA) in pentobarbital-anesthetized rats with or without bilateral vagotomy and in conscious saline-pretreated rats. Values are means of changes expressed as a percentage of baseline values. Vertical bars indicate SEM (5-6 rats per group). The dose-hypotensive and dose-tachycardiac response curves for conscious rats pretreated with saline (from Figure 2) were included here for comparison with those for intact, anesthetized rats. ^*∗*^ *p* < 0.05 by Dunnett's test with respect to basal values. ^#^ *p* < 0.001 by two-way ANOVA with respect to intact or bivagotomized, anesthetized rats.

In anesthetized rats, average baseline values of MAP and HR before any treatment were 106 ± 3 mmHg and 343 ± 13 beats/min, respectively (pooled data from 11 rats). Increasing bolus doses of EOCA (1–20 mg/kg) evoked immediate and dose-dependent decreases in MAP (*p* < 0.001, Figure 1(a)), without significant changes in HR (Figure 1(b)). Hypotensive responses to EOCA occurred within 10–12 s after the injection and became significant at the dose of 1 mg/kg (Figure 1(a)). After all doses tested of EOCA, predose values of MAP were fully recovered within the first 1 min following EOCA injection. Bilateral vagotomy did not alter baseline MAP (118 ± 5 versus 102 ± 5 mmHg) but significantly increased baseline HR values (410 ± 23 versus 345 ± 20 beats/min, *p* < 0.05). Bivagotomy failed to enhance significantly the dose-dependent hypotension elicited by EOCA (1–20 mg/kg) (*p* > 0.05, Figure 1(a)) while it unmasked significant and dose-dependent bradycardia (*p* < 0.001, Figure 1(b)).

**Figure 2:**
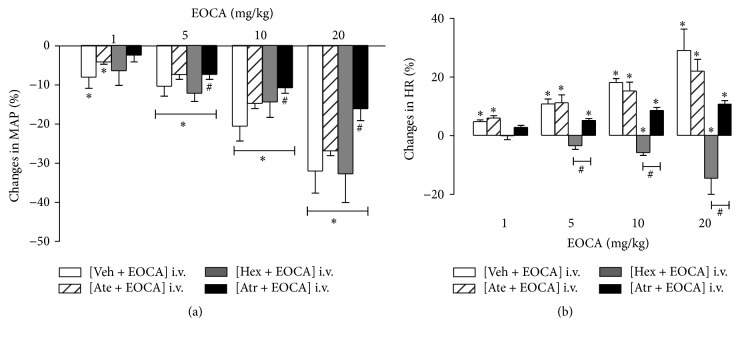
Maximal changes in mean aortic pressure (ΔMAP max, (a)) and heart rate (ΔHR max, (b)) elicited by intravenous (i.v.) injections of increasing bolus doses (1–20 mg/kg) of the essential oil of* Croton argyrophylloides* (EOCA) in conscious rats subjected to i.v. pretreatment with saline (Veh; 1 mL/kg), atenolol (Ate; 1.5 mg/kg), hexamethonium (Hex; 30 mg/kg), or methylatropine (Met; 1 mg/kg). Values are means of changes expressed as a percentage of baseline values. Vertical bars indicate SEM (5–7 rats per group). ^*∗*^ *p* < 0.05 by Dunnett's test with respect to basal values. ^#^ *p* < 0.05 by two-way ANOVA with respect to conscious, saline-pretreated rats.

Like in anesthetized rats, average values of MAP and HR before any treatment remained invariant and were 122 ± 2 mmHg and 367 ± 10 beats/min, respectively (pooled data from 28 rats), in conscious rats. Increasing bolus doses of EOCA (1–20 mg/kg) evoked immediate and dose-dependent hypotension (*p* < 0.001, Figure 2(a)), an effect that was associated with tachycardia that was also dose-dependent (*p* < 0.05, Figure 2(b)). Hypotension and tachycardia responses to EOCA occurred, respectively, within 6–8 and 10–14 s after the injection of EOCA and both became significant at the dose of 1 mg/kg (Figure 2). After all doses tested of EOCA, predose values of both MAP and HR were fully recovered within the first 1 min following EOCA injection.

In conscious rats, pretreatment with hexamethonium (30 mg/kg, i.v.) significantly (*p* < 0.01) decreased in baseline MAP (94 ± 8 versus 127 ± 6 mmHg) without affecting significantly baseline HR (368 ± 32 versus 320 ± 17 beats/min) values. However, pretreatment with methylatropine (1 mg/kg, i.v.) increased significantly (*p* < 0.05) the baseline HR (432 ± 27 versus 379 ± 24 beats/min) without affecting baseline MAP (130 ± 6 versus 124 ± 5 mmHg). Pretreatment with atenolol (1.5 mg/kg, i.v.) decreased significantly (*p* < 0.05) baseline HR (320 ± 21 versus 365 ± 26 beats/min) values but not those of MAP (121 ± 7 versus 127 ± 7 mmHg). Baseline MAP and HR values were significantly increased (154 ± 7 versus 118 ± 3 mmHg) and decreased (260 ± 25 versus 360 ± 17 beats/min), respectively, following pretreatment with L-NAME (20 mg/kg, i.v.).

**Figure 3:**
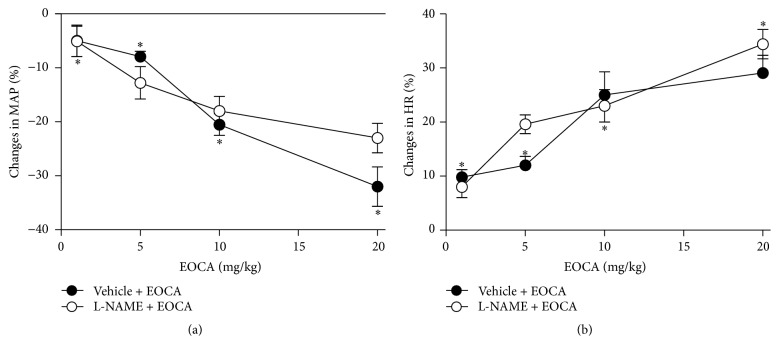
Maximal changes in mean aortic pressure (ΔMAP max, (a)) and heart rate (ΔHR max, (b)) elicited by intravenous (i.v.) injections of increasing bolus doses (1–20 mg/kg) of the essential oil of* Croton argyrophylloides* (EOCA) in conscious rats subjected to i.v. pretreatment with saline (Veh; 1 mL/kg) or L-NAME (20 mg/kg). Values are means of changes expressed as a percentage of baseline values. Vertical bars indicate SEM (6 rats per group). ^*∗*^ *p* < 0.05 by Dunnett's test with respect to basal values.

Pretreatment with hexamethonium did not only abolish (*p* < 0.05) the tachycardia elicited by EOCA but reverse it in significant (*p* < 0.05) bradycardia (Figure 2(b)) without affecting the hypotension (Figure 2(a)). However, both hypotension (Figure 2(a)) and tachycardia (Figure 2(b)) evoked by EOCA remained unaltered (*p* > 0.05) by pretreatment with either atenolol or L-NAME (Figure 3) while they were significantly reduced in rats pretreated with methylatropine (Figure 2, *p* < 0.001). In the latter animals, the remaining hypotension (Figure 2(a)) and tachycardia (Figure 2(b)) elicited by EOCA were still statistically significant with respect to baseline values (*p* < 0.05).

### 3.2. *In Vitro* Experiments

**Figure 4:**
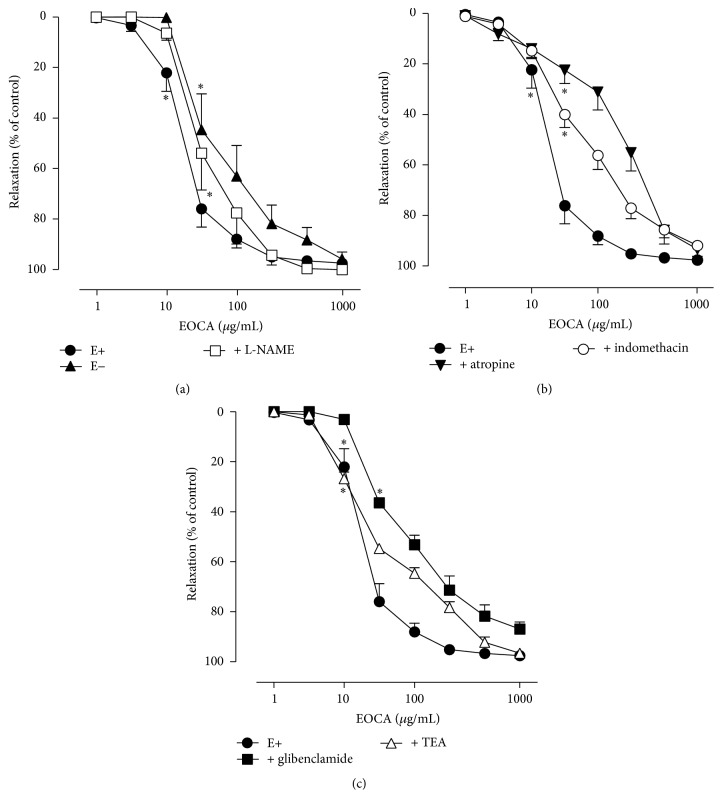
Effects of increasing concentrations (1–1000 *μ*g/mL) of the essential oil of* Croton argyrophylloides* (EOCA) on the contraction induced by phenylephrine (PHE, 1 *μ*M) in rat isolated thoracic aortic preparations with (*n* = 5) or without functional endothelium (*n* = 5) and those pretreated with L-NAME (100 *μ*M, *n* = 5) (a) or in preparations pretreated with atropine (1 *μ*M, *n* = 5) or indomethacin (10 *μ*M, *n* = 5) (b) or pretreated with glibenclamide (100 *μ*M, *n* = 5) or TEA (5 mM, *n* = 5) (c). Vertical bars indicate SEM. ^*∗*^ *p* < 0.05 by Dunnett's test with respect to basal values.

In aortic rings with intact endothelium, increasing concentrations of EOCA (1–1000 *μ*g/mL) inhibited the PHE-induced contractions in a concentration-dependent manner (*p* < 0.001). The first inhibitory effect of EOCA became significant (*p* < 0.05) at a concentration of 10 *μ*g/mL, while its maximal relaxation effect (2.54 ± 0.95% of the PHE-induced contraction) occurred at a concentration of 1000 *μ*g/mL (Figure 4). The IC_50_ (geometric mean [95% confidence interval]) value for EOCA-induced vasorelaxant effects was 25.31 [10.03–33.68] *μ*g/mL. These effects, which were reversible, were significantly reduced by removal of functional vascular endothelium (IC_50_ = 76.22 [36.38–116.30] *μ*g/mL) (Figure 4(a)) as well as by the previous pretreatment with atropine (IC_50_ = 197.20 [76.55–297.20] *μ*g/mL) (Figure 4(b)), indomethacin (IC_50_ = 90.60 [64.23–159.10] *μ*g/mL) (Figure 4(b)), or glibenclamide (IC_50_ = 64.46 [46.98–85.65] *μ*g/mL) in the bath (Figure 4(c)). The EOCA-induced vasorelaxation remained unaffected (*p* > 0.05) by the addition of L-NAME (IC_50_ = 41.75 [18.97–121.00] *μ*g/mL) (Figure 4(a)) or TEA (IC_50_ = 25.64 [19.87–59.48] *μ*g/mL) (Figure 4(c)) in the perfusion medium.

## 4. Discussion

The present study shows that i.v. treatment of either anesthetized or conscious normotensive rats with EOCA evoked immediate and dose-dependent hypotension which, in conscious rats, was associated with dose-dependent tachycardia. These cardiovascular effects, which are reported for the first time, are unrelated to EOCA's vehicle as it had no significant effects on either baseline MAP or HR values.

The role of the autonomic nervous system in the mediation of the cardiovascular effects of EOCA has been investigated in conscious rats. The hypotension and tachycardia were simultaneously reduced by i.v. pretreatment with methylatropine, indicating that a peripheral cholinergic mechanism is involved in their mediation. Blockade of ganglionic neurotransmission by hexamethonium did not alter significantly the hypotensive response to EOCA. This indicates that EOCA hypotension is independent of the presence of an operational central sympathetic neural drive to the vascular system, as was reported for essential oils belonging the* Croton* genus such as the EOCN [9, 10] and the EOCZ [15]. Furthermore, it is noteworthy that the decreases in MAP evoked by EOCA are more pronounced in diastolic than in systolic blood pressure which reflects reduced vascular peripheral resistance. Together, these findings raise the possibility that EOCA may decrease blood pressure through its vasodilator effects directly upon the vascular smooth muscle. The present* in vitro* findings of the concentration-dependent vasodilator effects EOCA in aortic rings preparations and those previously reported in mesenteric bed preparations [8] strongly corroborated such a hypothesis. As previously reported [8], chemical analysis of the sample of EOCA used in the present investigation identified twelve compounds, the major ones being spathulenol (26.6%), caryophyllene oxide (13.1%), *β*-elemene (12.1%), and *β*-caryophyllene (10.9%). It is possible that these constituents contribute, at least in part, to the vasorelaxant properties of the EOCA and further studies are needed to address this issue. Nevertheless, it is noteworthy that caryophyllene oxide has been also reported to induce an endothelium-dependent relaxation of aortic rings with an IC_50_ value ([2.5 (2.4–2.6)] *μ*g/mL) [25] that was higher than that reported herein with EOCA.

It might have also suggested that EOCA-induced vasorelaxant effects have been related to a putative toxic effect of this essential oil. However, two lines of evidences do not support this hypothesis. First, in our experimental conditions, all vasodilator responses to EOCA were reversible; that is, the sustained contraction induced by PHE was entirely recovered after EOCA removal. Second, our earlier finding of the very high LD_50_ (9.84 ± 0.01 g/kg) of EOCA administered* per os* in mice clearly demonstrated no acute toxicity [8]. In fact, according to a classification of the safety of essential oils used in aromatherapy [26], those exhibiting a LD_50_ value greater than 5 g/kg are considered as nontoxic to rodents.

In the present study an attempt was made to assess the mechanisms underlying the vasorelaxant effects of the EOCA. It is well established that vascular endothelium plays an important role in the control of vascular tone mainly through synthesis/liberation of endothelial-derived relaxing factors (EDRFs), such as NO and prostacyclin (PGI_2_) [27]. Accordingly, putative participation of the vascular endothelium in the mediation of EOCA-induced relaxation was investigated. Our results show that the mechanical removal of vascular endothelium partially, but significantly, reduced the vasorelaxant effect of EOCA as evidenced by the significant increase in the IC_50_ value of EOCA-induced reduction of the PHE-induced contractions. This suggests that the vasorelaxant effect of EOCA is partly mediated by an endothelium-dependent mechanism involving NO and/or prostacyclin release. The muscarinic receptor mediating relaxation of vascular smooth muscle is the M_3_ subtype and is located on the endothelial cells. Activation of this receptor evokes the release of EDRFs, mainly NO, which diffuses to the adjacent smooth muscle cells and stimulates the production of cGMP causing then the vasorelaxation. To assess whether EOCA-induced vasodilatation results from the stimulation of endothelial M_3_ receptors, aortic preparations were incubated with atropine. Under these conditions, vasorelaxation effects of EOCA were significantly reduced suggesting that they are partially mediated by the direct activation of endothelial M_3_ receptors. As EOCA-induced hypotension was also reduced by pretreatment with the peripheral muscarinic receptor antagonist methylatropine, one might have suggested that it would have been related to an active vascular relaxation mediated by an endothelial L-arginine/NO pathway through peripheral M_3_ muscarinic receptor activation. However, such a possibility seems unlikely since (i) the hypotensive response to EOCA was not changed by i.v. pretreatment with the NO synthase inhibitor L-NAME and (ii) the vasorelaxant effects of EOCA also remained unaltered by L-NAME.

Several studies have reported that NO is the most important EDRF responsible for the mediation of ACh-induced relaxation in rat aortic rings [28, 29]. However, some authors have reported that pretreatment with the cyclooxygenase (COX) inhibitor indomethacin attenuated the ACh-induced relaxation in rat [30, 31] or mice [32] aortic rings indicating that prostanoids may be involved. In the vasculature, especially in large conductance arteries, prostacyclin which is derived from the COX-2 is the most abundant prostanoid [33] synthesized in both vascular smooth muscle and endothelial cells [34]. In the present study, the EOCA concentration-response curve was shifted significantly to the right when aortic preparations were incubated with indomethacin suggesting the participation of prostanoids in the EOCA-induced vasorelaxation. Since the vasodilatory effect of EOCA was similarly attenuated by the removal of endothelium and by the inhibition of the cyclooxygenase, this may indicate that EOCA stimulate the release of prostacyclin from the endothelial cells. It has been demonstrated that prostacyclin synthesis by muscarinic agents such ACh is due to the activation of M_3_ receptors located on endothelial cells but not on the smooth muscle cells [35, 36].

Potassium channels play an important role in the regulation of vascular tone. Activation of these channels in the vascular smooth muscles causes membrane hyperpolarization leading to reduced intracellular Ca^2+^ and vasodilatation. Accordingly, vasorelaxation response to EOCA was studied in aortic ring preparations that have been pretreated with the nonselective K_Ca_ channel blocker, TEA, or the specific K_ATP_ channel blocker glibenclamide. Under these conditions, we found that vasodilatory action of EOCA was significantly attenuated by glibenclamide, but not by TEA, suggesting that it is closely associated with the activation of K_ATP_ channels. More studies are needed to further assess the mechanisms underlying the endothelium-independent vasorelaxant effects of EOCA.

In conscious animals, hypotensive response evoked by EOCA is associated with dose-dependent tachycardia. The following lines of evidence support the hypothesis that this tachycardia is of reflexogenic origin in response to decreased blood pressure. First, its maximum magnitude occurred (onset time of 10–14 s) later than the peak of the hypotensive effect (onset time of 6–8 s). Second, it was reversed into significant bradycardia in bivagotomized-anesthetized rats in which an apparent enhancement of the magnitude of EOCA-induced hypotension could be observed. Furthermore, EOCA-induced tachycardia was abolished when ganglionic blockade was achieved with hexamethonium, indicating that it is dependent upon the presence of an operational autonomic drive to the heart. The involvement of vagal component is corroborated by the finding that EOCA-induced tachycardia was reduced by methylatropine pretreatment. However, it seems that sympathetic activation is not involved as EOCA tachycardia remained unaltered by atenolol pretreatment although it was fully abolished by pentobarbital anesthesia. It is well known that the basal level of sympathetic nervous system activity is lower in anesthetized rats [37], and pentobarbital anesthesia depresses the baroreflex gain [38]. The finding that tachycardia response to EOCA is not only abolished but reversed into a bradycardic in either hexamethonium-pretreated conscious rats or bivagotomized-anesthetized rats may suggest that EOCA exerts opposite effects on HR. In conscious rats, the tachycardia component seems to predominate over the bradycardiac one.

In summary, the current study shows for the first time that i.v. treatment of both conscious and anesthetized rats with EOCA induces a dose-dependent hypotension, an effect that appears resulting from vasodilatory effects directly upon vascular smooth muscle rather than from withdrawal of sympathetic tone. EOCA-induced vasorelaxation seems partially related to muscarinic receptor stimulation, liberation of the endothelium-derived prostacyclin, and opening K_ATP_ channels. The tachycardia response recorded in conscious rats seems to be mediated reflexly through inhibition of vagal drive to the heart. The present findings may add a putative antihypertensive activity to the list of therapeutic uses for* C. argyrophylloides* in folk medicine. Further experiments using hypertensive rats are necessary to test this hypothesis.
